# Draft Genome Sequence of a Novel SAR11 Clade Species Abundant in a Tibetan Lake

**DOI:** 10.1128/genomeA.01137-14

**Published:** 2014-11-06

**Authors:** Seungdae Oh, Rui Zhang, Qinglong L. Wu, Wen-Tso Liu

**Affiliations:** a Department of Civil and Environmental Engineering, University of Illinois at Urbana-Champaign, Urbana, Illinois, USA; b State Key Laboratory of Marine Environmental Science, Xiamen University, Fuijan, China; c Institute of Marine Microbes and Ecospheres, Xiamen University, Fuijan, China; d State Key Laboratory of Lake Science and Environment, Nanjing Institute of Geography and Limnology, Chinese Academy of Sciences, Nanjing, China

## Abstract

SAR11 clade bacteria are abundant and play a key role in the nutrient cycles of marine and, presumably, inland aquatic environments. We report here the draft genome sequence of a novel species in the SAR11 cluster, reconstructed from a metagenomic data set obtained from a Tibetan lake.

## GENOME ANNOUNCEMENT

The SAR11 clade is a member of heterotrophic *Alphaproteobacteria* that are widely distributed in marine and inland aquatic ecosystems (1–3). While the SAR11 clade bacteria are abundant and play an important role in marine biogeochemical cycles, their ecological roles in lacustrine ecosystems remain relatively less characterized. Lake Qinghai represents the seventh largest lake on Earth, located on the Tibetan Plateau (4). Our previous 16S rRNA gene clone library analysis of the planktonic microbial communities obtained from the mountain lake discovered SAR11-like sequences, which were abundant (up to 19% of the total bacteria) and phylogenetically distinguishable from the freshwater subclade IIIb/LD12 lineage (5). Here, we report the draft genome sequence of the SAR11 clade species obtained from Lake Qinghai.

The draft genome sequence of the SAR11 clade species alphaproteobacterium strain QL1, was recovered from a metagenomic data set of a surface water sample taken from Lake Qinghai. The DNA was extracted as described previously (4, 5), and the Roche 454 GS-FLX sequencing technology generated 577-Mbp metagenomic sequences. The metagenomic data set was trimmed based on a Phred quality score cutoff of 10 using SolexaQA2 (6) and assembled using Newbler 2.8. Binning of the assembled contigs was carried out based on metagenomic read coverage, tetranucleotide frequency, and the occurrence of unique marker genes using MaxBin (7), which generated 99 clustered contigs. Searching the 99 contigs against all bacterial and archaeal genome sequences available in the GenBank database (as of January 2014 [ftp://ftp.ncbi.nih.gov/]) using BLASTn (8) showed best hits to “*Candidatus* Pelagibacter” IMCC9063 (9). Metagenomic read recruitment on the 99 contigs using BLASTn showed >95% nucleotide sequence identity with >20× coverage. The sequence composition-based binning, the sequence homology search, and the metagenomic read recruitment collectively indicated that the 99 contigs represent well the draft genome sequence of the single-species population.

The draft genome was 952,704 bp, with 31.4% G+C content. Gene prediction and functional annotation on the draft genome were performed using the RAST server (10) and the NCBI Prokaryotic Genomes Automatic Annotation Pipeline (PGAAP) (http://www.ncbi.nlm.nih.gov/genomes/static/Pipeline.html). The draft genome contains a total of ≥870 protein-coding genes (>300 bp), a 16S rRNA gene, a 23S rRNA gene, and 20 tRNA genes. Searching the 16S rRNA gene against a reference rRNA sequence database (GenBank) using BLASTn revealed the highest sequence identity to “*Ca.* Pelagibacter” IMCC9063 (98% identity) in subclade IIIa. Searching the 16S rRNA gene against the nucleotide sequence collection database (GenBank) using BLASTn revealed 100% sequence identity to uncultured bacterium clone sequences obtained from the Chesapeake Bay and 99% sequence identity to those obtained from the Baltic Sea, Delaware Bay, and Lake Nam Co (5, 11). These results highlighted the global distribution of the QL1-like species in both lacustrine and marine ecosystems. We are currently exploring the gene content and genomic variability of the draft genome compared to those of other SAR11 clade members.

### Nucleotide sequence accession numbers.

This whole-genome shotgun project has been deposited at DDBJ/EMBL/GenBank under the accession no. JPLS00000000. The version described in this paper is version JPLS01000000.
